# Using team science in vascularized composite allotransplantation to improve team and patient outcomes

**DOI:** 10.3389/fpsyg.2022.935507

**Published:** 2022-09-06

**Authors:** Joan M. Griffin, Cassie C. Kennedy, Kasey R. Boehmer, Ian G. Hargraves, Hatem Amer, Sheila G. Jowsey-Gregoire

**Affiliations:** ^1^Division of Health Care Delivery Research, Mayo Clinic, Rochester, MN, United States; ^2^Robert D. and Patricia E. Kern Center for the Science of Health Care Delivery, Mayo Clinic, Rochester, MN, United States; ^3^Division of Pulmonary and Critical Care, Mayo Clinic, Rochester, MN, United States; ^4^The William J. von Liebig Center for Transplantation and Clinical Regeneration, Mayo Clinic, Rochester, MN, United States; ^5^Knowledge and Evaluation Research Unit, Mayo Clinic, Rochester, MN, United States; ^6^Division of Nephrology and Hypertension, Mayo Clinic, Rochester, MN, United States; ^7^Essam and Dalal Obaid Center for Reconstructive Transplant Surgery, Mayo Clinic, Rochester, MN, United States; ^8^Department of Psychiatry and Psychology, Mayo Clinic, Rochester, MN, United States; ^9^Department of Surgery, Mayo Clinic, Rochester, MN, United States

**Keywords:** vascularized composite allograft (VCA), team science, qualitative study, case study, transplant

## Abstract

Reconstructive allografts using Vascularized Composite Allotransplantation (VCA) are providing individuals living with upper limb loss and facial disfigurement with new opportunities for a sensate, esthetically acceptable, and functional alternative to current treatment strategies. Important research attention is being paid to how best to assess and screen candidates for VCA, measure optimal patient outcomes, and support patient adherence to lifelong behaviors and medical regimens. Far less attention, however, has been dedicated to the team science required for these complex VCA teams to form, prepare, and provide the highest quality clinical and psychosocial care to those receiving VCA. VCA teams are unique in that they require specialized team members whose scope of practice may not otherwise overlap. The team also needs to constantly negotiate balancing patient safety with multiple risks throughout the transplant process. This study aimed to elucidate the team science needed for this highly innovative and complex area of medicine. Using in-depth qualitative interviews with 14 VCA team members and observations at team meetings, we found that careful consideration of team composition, team structure, and organizational commitment (e.g., local culture and team values; investment of resources) influences team performance and patient outcomes, but that to be efficient and truly effective, teams need to commit to developing processes that foster collaboration. These processes are action-oriented (e.g., communication, leadership), strategic (e.g., planning, training) and interpersonal (e.g., conflict management, trust building). Dedication and commitment to team science allows teams to manage conflict under stress and exercise ways to leverage strengths to provide optimal performance or patient psychosocial and clinical outcomes. This study can provide insight into quality improvement efforts for VCA teams and guidance for other transplant programs that wish to consider expansion into VCA.

## Introduction

The first reported hand transplantation was performed in Lyon, France in 1998 (Dubernard et al., 1999). Since then, more than 120 hand transplants in 76 patients and 37 face transplants have been performed, with reasonable functional and esthetic outcomes for optimally selected patients (Thuong et al., 2019). These reconstructive transplants, using Vascularized Composite Allografts (VCA), are providing some persons living with upper limb amputation and facial disfigurement with new opportunities for a sensate, esthetically acceptable, and functional alternative to current treatment strategies (Kaufman et al., 2019). With advances in immunosuppression, surgical and medical techniques, and technology, these transplants while complex and associated with long term risks of immunosuppressive medications, are no longer considered experimental, but feasible. The field, therefore, is at a juncture. These procedures are possible, but they are not yet part of the standard options routinely presented to eligible patients. The psychosocial and ethical challenges associated with VCA and how those challenges are translated into VCA care models and treatment standards are persistent questions. These challenges include how best to identify and select ideal candidates, communicate short- and long-term consequences, support short- and long-term rehabilitation goals, and define and attain optimal clinical and psychosocial outcomes. To optimize patient outcomes and satisfy requirements likely needed for insurance coverage and regulatory needs, clear methods to overcome these challenges are required. Further, these strategies must be considered within the ethical context and assumptions of VCA improving quality of life, not, like other transplants, to save or sustain life (Caplan et al., 2019).

With VCA opportunities still limited to relatively few academic health centers—18 hand and 17 face transplant programs as of 2019—much of the research to fill these gaps is underway with the highly specialized teams that currently have VCA programs (Henderson, 2019). One important area of inquiry with less attention, however, is the team science required to form these complex, transdisciplinary VCA teams and programs that develop and shape models of care and influence the local approach to transplant. Cultural understanding of these highly specialized teams is important, as it is a shaping factor in the ways in which patient-level concerns and challenges noted above are met. Such an understanding can provide insight as other transplant programs consider expansion into VCA and more generally, to advance the understanding of barriers and facilitators required for transdisciplinary teams practicing in highly innovative and complex areas of medicine, areas where the benefits for a patient’s quality of life needs to be constantly evaluated with safety and risks.

We interviewed healthcare providers as part of a larger study aimed at understanding the values, attitudes, and expectations of treatment for people with facial disfigurement and upper extremity limb loss. These members of the healthcare team were engaged in discussions about possible treatment options, candidate selection, VCA preparation, surgery, patient recovery and rehabilitation, and in VCA team meetings. One major theme that emerged in the interviews was consistent with definitions of team science: how team members organically formed and came to work collaboratively toward a common goal of establishing a VCA center and providing optimal outcomes for patients. Using a case study approach, we examined this “team science” theme.

## Materials and methods

### Study case

This study uses a qualitative case study methodology. Yin (2003) defines a case study as an empirical research activity that examines a specific situation within a real-life context. Case studies typically include a limited number of informed individuals with detailed understanding of the case or the context of the case. Qualitative methods for team science have been endorsed because they allow for identification of essential factors, a close proximity to processes (Yin, 2003). When conducted with teams in extreme settings or exceptional circumstances, such as VCA, they provide in-depth findings that can inform other teams in similar circumstances on models of best practice (Solis et al., 2016).

For this case we focused on a VCA team that has successfully completed one VCA transplant, but is actively assessing potential candidates for future procedures.

#### Study design

From March 2019 to March 2021, we conducted 14 unstructured interviews with the transdisciplinary team that included surgeons, transplant physicians, a psychiatrist, transplant nurse coordinator, social worker, ethicist, and prosthetists. This study was approved as minimal risk by the Institutional Review Board (IRB 18-006889) and oral consent was obtained from all study participants.

#### Source of participants

We used a purposeful “maximum variation” sampling strategy to capture differences between providers (Patton, 2002). Figure 2 shows the composition of the VCA team. Spheres in green represent clinical team members, and spheres in blue represent institutional or administrative team members. Representatives from each of the clinical team spheres were included in interviews.

VCA team leaders representing the surgical team, transplant team and bioethics (indicated by the overlapping green spheres at the center in Figure 2), were interviewed. They identified additional key stakeholders for interviews from other spheres. These included physicians and nurses, social workers, physical therapists, transplant coordinators, and prosthetists and protheses fitters involved in the VCA team. Names and contact information for additional key stakeholders were obtained through the VCA team leadership and through snowball sampling. Participants were contacted by either the study coordinator or the principal investigator *via* email to explain the study purpose, consent script, and to request participation. If no response to the initial email was received, one additional follow-up email was sent. A study coordinator contacted interested providers to schedule interviews with one of two interviewers. Both interviewers were experienced Ph.D. trained researchers who had not previously worked with any of the providers. All team members approached (*n* = 14) agreed to be interviewed and completed interviews. Recruitment continued until saturation of information on treatment options, candidate selection, and VCA preparation.

#### Source of data

The interview guide was initially unstructured, but aimed to understand the team’s interactions and perspectives on VCA education, candidate selection, and once a candidate was listed, the pre- and post-VCA preparation and treatment discussions with patients and families. With every subsequent interview, the guide was modified to become semi-structured, and reflected new areas of inquiry or greater concentration on topics that were emerging as critical points for understanding. For example, with little prompting, those interviewed early in the process discussed at length the importance of collaboration and the development of the study team as it related to patient education and outcomes. Probes about team science, therefore, were integrated into the interview guide over time.

All interviews were digitally recorded and transcribed verbatim and then de-identified (removing names and other identifiable features). After each interview, case-based memos were created that captured ideas and compared accounts with other participants. Recorded interviews were transcribed verbatim and stored on a secure server for analysis in NVivo (2020).

For team meeting observations, qualitative analysts attended meetings and took notes. Notes were uploaded into Nvivo for analysis. Meetings included 63 invited team members, with attendance varying depending on the case and agenda.

#### Data analytic approach

The research team used a three-phased thematic content analysis to understand provider attitudes, beliefs, and experiences about the VCA process (Patton, 2002; Bradley et al., 2007). First, three research team members (JG, KS, and CK) independently read the interviews multiple times to become immersed in the data and then began an initial set of codes that captured key concepts from the data (e.g., team collaboration). Second, JG, a health services researcher with qualitative and mixed methods expertise, and CK, a solid organ transplant physician with qualitative research experience, further analyzed the transcripts, met regularly to expand, and refine code development, and create definitions for each code. With the review of each subsequent transcript, codes were refined (e.g., defining roles, coordination, program investment) to reflect a deeper understanding of the team science theme. Coding decisions were developed by consensus and documented to provide a clear audit trail on the origin of the codes. After finalizing the code book, JG and CK then coded each transcript and applied codes line by line back to the transcripts. Third, codes were then organized into sets of codes specific to team science and analyzed for relationships across that set of codes.

## Results

**FIGURE 1:**
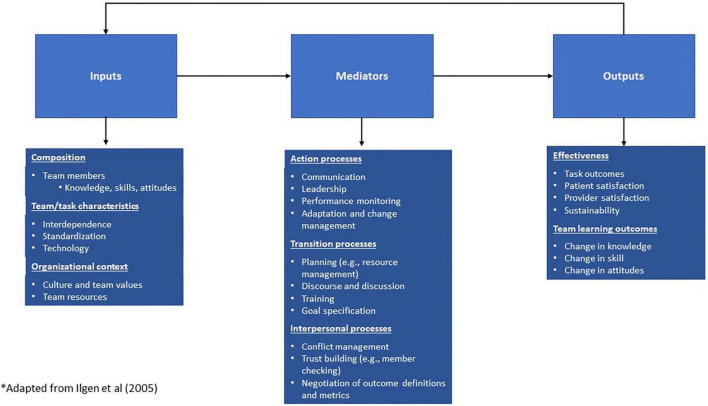
Input-mediator-output-input framework for VCA team science.

One major theme across interviews and observation data was how providers worked together to develop and create an environment to promote successful outcomes when stakes are high, and surgeries are infrequent. As shown in Figure 1, we coded this theme as “team science” and as we analyzed sub-codes, we recognized the alignment of our subcodes with the constructs from the Input-Mediator-Output-Input framework, the dominant framework in team science that describes dynamic, causal relationships. It details affective, cognitive, and behavioral processes that affect team performance, where inputs and mediators explain variability in team output and viability, and output, in turn, affects the next iteration of inputs (Ilgen et al., 2005). Using this framework assists in helping to better understand the team’s experiences and their intentional and unintentional strategies that form the transdisciplinary team and refine team processes for VCA transplants.

**FIGURE 2:**
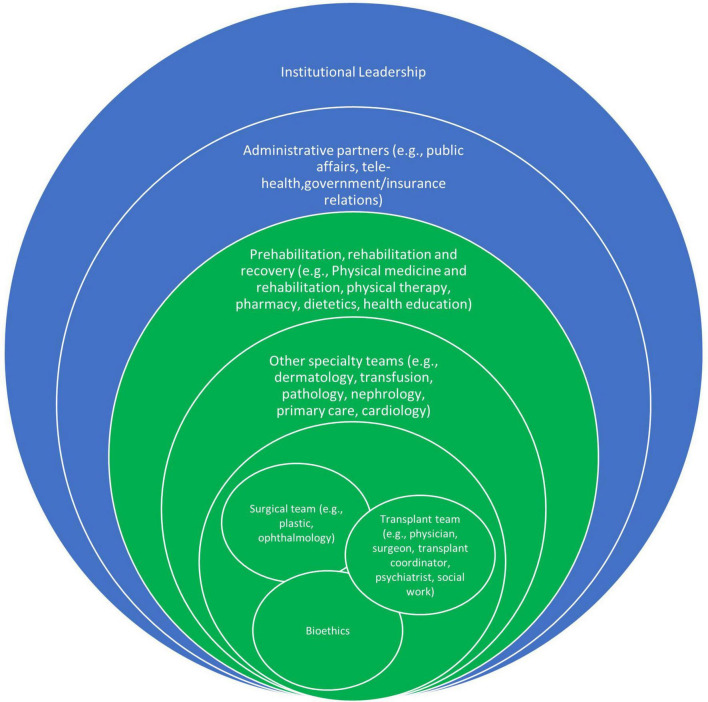
VCA team composition.

### Inputs

Inputs included team organizational commitment (e.g., local culture and team values; investment of resources), team structure, and composition, all of which are considered to influence team performance and patient outcomes. Participants described teams by their level of engagement and their unique skills. More often, participants described their role on the “teamlet” (Bodenheimer and Willard-Grace, 2016) or the smaller core teams. There was a deep understanding, however, that the teamlet was part of a larger integrated team and a commitment to that team. Participants considered their organization’s cultural values as a key element of how and why the team formed. Institutional and departmental leadership financially invested in developing and sustaining the program, encouraged learning from existing external programs, provided resources that allowed for leaders in transplant and plastic surgery—teams that typically do not share cases—to collaborate and initiate a new model of care. Figure 2 is a synthesis of team roles and the described levels of team participation, with the inner circle having primary leadership roles, greatest number of responsibilities, and most interdependence.

The VCA team was led by transplant and surgical leaders who drew from existing teams and resources. Notably, the VCA program did not maintain any personnel whose sole employment duties pertained to the VCA program. Salary and benefits were maintained by their primary position and a proportion of their time and expertise was allotted to the VCA program. Further, the VCA team used space in an established solid organ transplant center. Thus, the VCA program did not require infrastructure funds at its inception.

The team members entered the VCA program with established training and expertise in their field and adapted this experience to serve the VCA program. Some of the team members worked in multidisciplinary solid organ transplant teams but many were new to transplantation. Team members without transplant experience reported seeking out workshops or shadowing opportunities elsewhere to build familiarity with transplant practice. Similarly, team members with transplant backgrounds sought opportunities to understand the needs of patients cared for by the reconstructive surgery practices.

Team composition evolved with purpose over time. Leaders chose members for their teamlets based on previous experience, knowledge about transplant or surgical processes, and team members were added as needs emerged. For example, bioethicists were brought on early to help dissect ethical issues of offering non-life saving transplants, navigate candidate selection issues (e.g., ethics of offering bilateral vs. unilateral hand transplantation), and as additional selection and evaluation issues emerged, the bioethicists were integral members of the core VCA team.

*“The transplant center has an integrated transplant practice, and although all are solid organs or BMT [bone marrow transplant], they are under the same roof, so we should try to merge those [areas of expertise] together’*… *And if you look at it, you need surgeons to be able to do the procedures. You need physical therapy, like a hand therapist, and we need physiatry because physiatry helps with the rehabilitation. Then you have the amputee clinic because the amputee clinic manages individuals who are amputees, so their expertise was important*…*So what you’re doing there is trying to make sure that, for the normal entities that take care of a particular population, you have representation.” (VCA team member, #1)*

*“[From conversations with another VCA team]*…*they viewed their team as like a spider web, and there were all these nodal points of information that then would go up through the web. They felt in their team, there was one person who kind of was the spider who caught all the information. And they proposed that with every team, there’s one person who kind of functions in that role*…*in our team, it isn’t one single person who does that. I think because of a shared electronic medical record and the [institutional] tradition of us all working in a very close fashion, team meetings, frequent consultations, we’re not all separated*…*.”[VCA team member, #2]*

“…*I recall that the VCA team began to form as more and more prospects for transplantation began to develop. My primary involvement was to be a part of all of those meetings. I attended all of the VCA meetings and, as a result of that, my council was often solicited with respect to clinically relevant issues related to VCA.” (VCA team member, #3)*

### Mediators

Mediators included the development of action-oriented (e.g., communication, leadership), strategic (e.g., planning, training) and interpersonal (e.g., conflict management, trust building) processes. These processes shaped how the team considers potential candidates, functioned, and managed conflict under stress, leverageed individual strengths, and prepared for optimal performance. Participants highlighted two key strategies that helped to establish and sustain a highly functional and prepared team. The first strategy was the structure of scheduled meetings for assessment and evaluation of potential VCA candidates. The primary purpose for these ongoing meetings was to review a potential candidate’s case by evaluating the medical criteria for transplant, psychological assessments, and a patient’s psychosocial well-being and available social support. Team members presented evaluation reports for potential candidates. Although evaluation of medical and transplant criteria were relatively straight-forward, the psychological and psychosocial evaluations were more challenging and ambiguous. Team leaders welcomed differing opinions about the evaluation conclusions from any team member and when concerns were raised, the team had a thorough and deliberate discussion. If deemed appropriate, additional information about that concern was collected or new team members with expertise about that concern were included. Based on participant interview data and observation notes, the unintentional consequence of these meetings was action-oriented and interpersonal processes that built trust, collaboration, and conflict resolution among the team. Team meetings led to frank discussions about selection criteria for candidacy, especially the psychosocial readiness of candidates both before and after listing them for transplant. These meetings continued even when there was not an active candidate listed for transplant or when surgery preparation or performance was imminent, allowing teams to continue to form and develop cohesion. All disciplines involved in VCA were included and participants remarked that leaders flattened hierarchies, thus reinforcing a sense of each discipline’s critical contribution to the overall team and trust across disciplines. They emphasized individual expertise and the mutual contribution that each expert brought to the process. After a candidate was listed these thorough and candid discussions promoted a collective commitment and reinforced the team’s belief that VCA was a viable, safe, and reasonable option that would benefit the selected candidate.

*[regarding leadership in team meetings] “*…*taking turns in speaking so that everybody contributes about the same number of minutes to the discussion. It’s this very egalitarian way, so everybody feels comfortable enough to say something. There’s no top-down leadership style*…*it’s easy within medicine for there to be a hierarchy. But this just doesn’t feel that way. It really feels like you are truly all equal contributors to a team.” (VCA team member, #4)*

*[team processes regarding selection and evaluation]“*…*we began early on, I believe, as a group to formulate a sense of what risk meant to us and what risk meant to those that we assumed were so vulnerable. These terms all began to take on different shades. I think, prior to my involvement [on the VCA team], I would have never had problems articulating what I thought was consistent with the word “risk,” but that changed substantially over time, and I believe it did for my colleagues, too. So, we were actively growing together and juggling these very abstract terms, all of which were superimposed on a very intense sense of beneficence and commitment to the welfare of potential recipients.” (VCA team member, #2)*

*[challenges with selection] “*… *if there were a patient who you felt some conflict with or some incompatibility up front that that would be very difficult to work through as a team and to have that patient have a good outcome. (VCA team member, #4)*

*“[the team process for VCA preparation]*…*it really is like launching a space rocket, that you have to have many, many, many, many, many, many checks and balances. And if the safety light is lighting up, you gotta stop everything, and you’ve gotta go back and check and not proceed until all systems are go. And if all systems aren’t go, then you shouldn’t go. I think [the recipient] did get that message, that that’s how we would proceed because it’s such an enormous responsibility to [the recipient] to do it right*…. *You’ve gotta have everybody kind of matched and moving in a concerted way together, and all your harnesses have to be buckled up and secured before you can really proceed safely. (VCA team member, #2)*

The second key strategy was VCA team practice sessions in preparation for when a suitable VCA candidate was selected. Team members volunteered their time over 50 Saturdays to create protocols, practice transplant techniques, safety plans, hand-offs, and transitions in the transplant process (Amer et al., 2018). These practice sessions helped form strategic, action-oriented, and interpersonal processes for the team. The team developed a commitment to creating and refining optimal procedures and built trust and cohesion. They formed a collective belief in their skill and efficacy, along with a shared mental model of the VCA process. Practice sessions led to better role clarification, development of contingency plans, and strategies for addressing interpersonal conflicts and administrative barriers. They simulated interpersonal and leadership communication patterns and highlighted areas where additional team coordination was needed. They also informed the need for additional team training, administrative task planning (e.g., blocking operating rooms at short notice), and clarification of necessary procedures outside of the specific VCA procedure (e.g., media relations, communication with and support of donor family). Practice sessions were adapted to the VCA candidate’s case once selected.

*(“Interviewer: Were the Saturday sessions everyone just saying “I want to be a part of this” or was it volunteer? How did that happen?) “I mean it was just actually building the team over time. At first it was a small group, two, three people going into the cadaver lab for maybe five or six times or seven times. Trying to decide*…*trying to figure out the basics. And then saying, ‘You know what, here’s the patient’s defect, let’s think through how we would do it’, and then we thought through that and then once we decided, you know this is a pretty reasonable protocol for this particular defect for our patient. Then we started building the team and rehearsing and of course, still during the rehearsal you are still modifying and improving things.” (VCA team member, #5)”*

“…*they [the VCA team] practiced and practiced and practiced on cadavers until they almost could do it by memory. They knew every step, every part of step, and it still took, I think he said it took 52 hours, but like an orchestra, everybody came in. I like the analogy of an orchestra. You may be the first violinist but when it’s the oboe part, you sit there quietly and let the oboist play. And you can’t just say ‘Well, I’m the first violinist, you’ve got to be quiet. I’m going to play.’ You play when it’s your turn. You come in and do your job and back off and let somebody else do their job. Everybody knows their part. That’s what the team’s all about. There’s nothing special about anyone of us. But as a team we do a pretty good job” (VCA team member, #6)*

*”I’ve become interested in how do you build a successful team*…*it is like kind of lightning in a bottle. When you have it, it’s great. Trying to recreate it is really difficult. Certainly the anatomy lab sessions help tremendously, spending so many Saturdays together working together on this common project*…. *So I think a huge amount of the credit goes to [the surgical leader] and then, yeah, forming those bonds in the anatomy lab, working towards that common goal. And then it also helps when you have a great outcome, too. It just bonds the team further together.” (VCA team member, #4)*

### Outputs

Outputs included team learning, patient selection and transplant outcomes, and sustainability of the team and its processes. As noted, team participants had a collective commitment to assuring optimal patient outcomes, regardless of whether a potential candidate progressed to VCA. In addition to the overall success of the transplant, each teamlet had their own individual markers of success for VCA that were associated with their assigned task and area of expertise.

“…*afterwards he was able to smile on both sides. Because that’s – that’s success. Success is getting the smile to – you know it’s not perfect but to look like a smile. And he’s able to close his mouth.” (VCA team member, #5)*

“…*as surgeons, we would wanna see function, you know. We would wanna see sensation. (VCA team member, #7)*

*“If I was to talk to a new team forming*,…*[I] would suggest to them that they get a sense of the rapport-building abilities of the other team members. How do the physician, medical director, and the key physical therapy staff*…*they have to be people who are experienced and known to be able to work well with patients. This is something you’ve gotta really have some years of clinical experience to do and be vetted by your surgical departments before you move forward. (VCA team member, #2)*

More elusive to the team, however, was building consensus on the optimal approach for determining at each step of the VCA process (assessment/evaluation, procedure, recovery, and rehabilitation) how the expectations and goals of patients are being met. This reflection led to a new cycle of inputs and mediators for the VCA team to consider. These included capturing patient expectations early, assessing alignment of early expectations with procedural realities, and refining tailored educational and rehabilitation efforts to meet those expectations.

*“[With VCA]*… *I’m definitely thinking about for a future candidate*…*I think that’s where some peer mentoring from other patients is so important because there are just so few people who’ve ever gone through this.” (VCA team member, #2)*

*“They [VCA recipients] don’t want to [only] get their hand back so that they can go back to work, they want their hand back so that they can touch the face of their loved one. So that they can hold the hand of their loved one*…*it’s holding hands and feeling the skin of their hands. [The hand] kind of is an intimacy organ, you could say*… *they sustain these primary family relationships. So after I started having these new thoughts [of hands as intimacy organs] I started thinking ‘now I really haven’t thought this through the right way.”’ (VCA team member, #8)*

## Discussion

The growing recognition that complex problems are often best served by cross-disciplinary expertise and intense collaboration has propelled the evolving field of team science. Transplant surgery, including VCA, relies on transdisciplinary teams, collaboration across specialties, and coordination of processes to identify, assess, and list potential transplant candidates, and prepare for the transplant, surgery, recovery, and rehabilitation (Costanzo et al., 2010; De Pasquale et al., 2014; Cajita et al., 2017). It is surprising, therefore, that little research has been conducted to describe the team science of transplant, and how a transplant program’s composition, formation, and interactions might influence the effectiveness of patient outcomes and team effectiveness. In that regard, our study is relatively novel and provides an understanding of the components of effective teams and some of the ongoing challenges in VCA.

In our study we found that VCA teams require specialized team members whose scope of practice may not otherwise overlap. Team members need to negotiate about balancing patient safety, psychosocial well-being, and multiple risks throughout the transplant process. The VCA team in our case study required: (1) a significant investment of institutional, medical and financial resources to form; (2) multiple team members with specific expertise and highly advanced skills; (3) careful and precise protocols to reduce the risk for mistakes/errors when multiple external factors are out of the team’s control; and (4) significant practice and simulation prior to these relatively rare surgeries in order to improve precision of technique and to build role definition, trust, and a collective mental model of metrics for success. The team’s self-reflection on their care processes identified gaps in how patient expectations were assessed, how they defined success, and how their expectations and definitions of success changed throughout the transplant process.

Our findings are consistent with other studies in team science. Institutional culture and values, for example, especially a culture of cooperation where shared ideas across disciplinary boundaries are communicated and cultivated, is a key indicator to academic innovation (Lee and Jabloner, 2017). As we found in our study, team effectiveness has been shown to hinge on team member familiarity and social cohesiveness, which builds over time and through shared experiences (Stokols et al., 2008). Finally, teams have been shown to perform best when there is cooperation and interdependence of a team’s tasks and rewards (Stokols et al., 2008).

Our study does have limitations to consider. First, our examination of team science emerged organically from interview and observation data and was not the original intention of the study. Targeted questions about team science, therefore, were not asked of participants. Future research can build from our findings by developing intentional questions about how teams function and the effect that a team’s cohesion has on patient outcomes. Based on our findings we provide potential questions for others to consider when evaluating their own teams in Supplementary Figure 1. Second, the VCA team studied had, to date, only completed one VCA surgery. Data from teams that have a longer record of completed transplants may result in different foci or mediators that affect outcomes. Capturing data from an established team with fewer transplants, however, may provide acute insights into the challenges of early formation of a VCA team or for programs considering expansion into VCA. Third, only two sources of data were used: participant interviews and observations. Additional data sources may have resulted in either a deeper understanding of team dynamics or additional characteristics of the team not found in the available data. Future research may benefit from using sensors to capture data, such was done by Rosen et al. (2018) or by using other methods, such as video reflexive ethnography (Carroll et al., 2018) where team members review video of their teamwork in order to reflect on opportunities for improvement. These alternative methods may be especially appropriate for specific stages of VCA, such as surgery and recovery and to inform later phases of team development, reflexivity, and sustainability. Finally, understandably, our data had a greater emphasis on the impact of team science on surgery preparation and performance, and less on how team science could directly affect candidate selection and indirectly, recipient outcomes. In order for teams to reflect on process and system improvement, future research could include ethnographic investigations that include different team approaches from candidate identification through final treatment choices. Future research could also focus on how teams build and maintain cohesion and manage attrition during the less intense stages of candidate identification and assessment or when surgery preparation and performance is not imminent.

In spite of these limitations, findings from this study may be especially helpful for other programs considering how to form a team to expand into VCA or improve existing teams, but they may also be of interest to other highly complex, transdisciplinary teams who perform procedures that are not life-saving, but rather aimed at improving patients’ quality of life, such as gender reassignment surgery and bariatric surgery. Based on our findings, these newly forming teams should consider selecting highly experienced, compatible members to participate; an open, methodical, and collaborative approach to leadership; development of team building processes that provides opportunities to practice and build trust; team consensus on definitions of team and patient success; and strategies for self-reflection and evaluation.

## Data availability statement

The original contributions presented in this study are included in the article/Supplementary material. Further inquiries can be directed to the corresponding author.

## Ethics statement

The studies involving human participants were reviewed and approved by the Mayo Clinic Institutional Review Board. Written informed consent for participation was not required for this study in accordance with the national legislation and the institutional requirements.

## Author contributions

JG: study conception and design and initial draft of the manuscript. JG and KB: data collection. JG and CK: analysis and interpretation of the data. JG, CK, KB, IH, HA, and SJ-G: critical revisions. All authors reviewed the results, edited early versions of the manuscript, read, and approved the final version of the manuscript.
