# Supplementary figures and images for: Using team science in vascularized composite allotransplantation to improve team and patient outcomes
Source: Front Psychol. 2022 Sep 6;13:935507. doi: 10.3389/fpsyg.2022.935507 (PMC9486538; doi:10.3389/fpsyg.2022.935507)

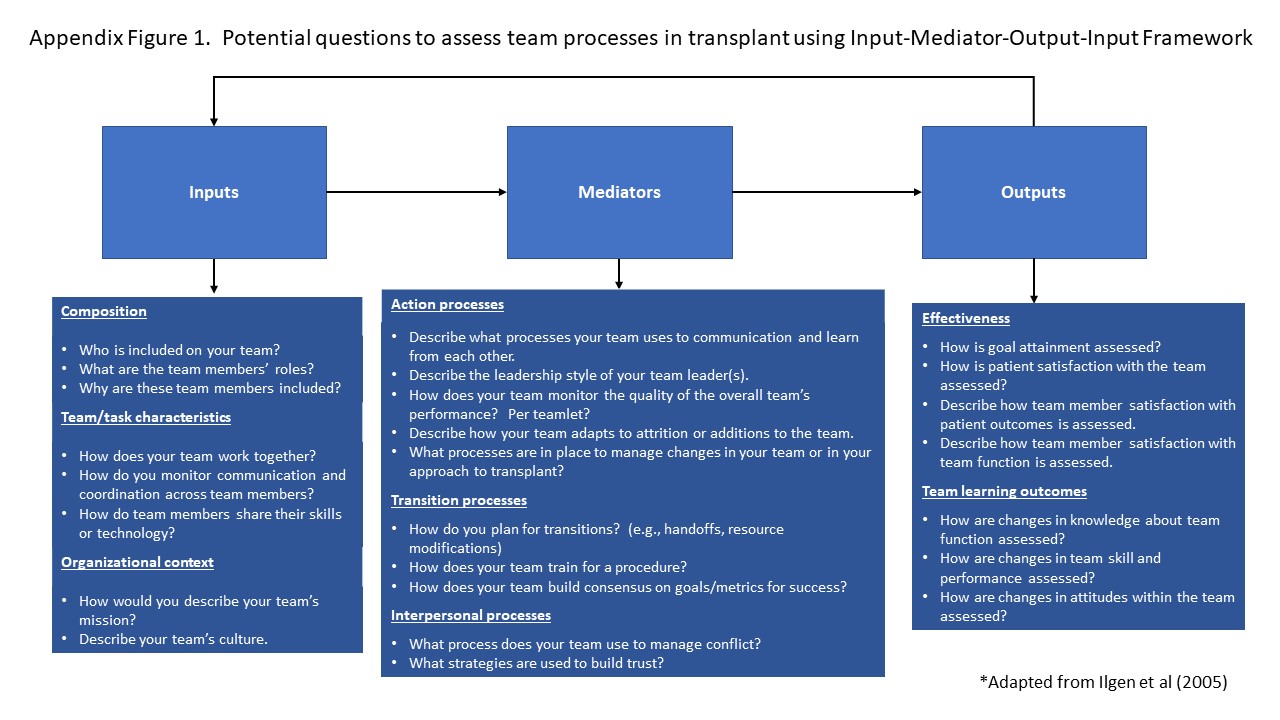

Supplement: Supplementary file 1 [file Image_1.JPEG]
